# Rates of New Persistent Opioid Use After Vaginal or Cesarean Birth Among US Women

**DOI:** 10.1001/jamanetworkopen.2019.7863

**Published:** 2019-07-26

**Authors:** Alex F. Peahl, Vanessa K. Dalton, John R. Montgomery, Yen-Ling Lai, Hsou Mei Hu, Jennifer F. Waljee

**Affiliations:** 1 Department of Obstetrics and Gynecology, University of Michigan, Ann Arbor; 2 Institute for Health Policy and Innovation, University of Michigan, Ann Arbor; 3 Program on Women’s Healthcare Effectiveness Research, University of Michigan, Ann Arbor; 4 Department of Surgery, University of Michigan, Ann Arbor; 5 Michigan Opioid Prescribing Engagement Network, Department of Surgery, University of Michigan, Ann Arbor

## Abstract

**Importance:**

Research has shown an association between opioid prescribing after major or minor procedures and new persistent opioid use. However, the association of opioid prescribing with persistent use among women after vaginal delivery or cesarean delivery is less clear.

**Objective:**

To assess the association between opioid prescribing administered for vaginal or cesarean delivery and rates of new persistent opioid use among women.

**Design, Setting, and Participants:**

This retrospective cohort study used national insurance claims data for 988 036 women from a single private payer from January 1, 2008, to December 31, 2016. Participants included reproductive age, opioid-naive women with 1 year of continuous enrollment before and after delivery. For participants with multiple births, only the first birth was included.

**Exposures:**

Peripartum opioid prescription (1 week before delivery to 3 days after discharge) captured by pharmacy claims, including prescription timing and size in oral morphine equivalents. Multivariable adjusted odds ratios were estimated using regression models.

**Main Outcomes and Measures:**

Rates of new persistent opioid use, defined as pharmacy claims for 1 or more opioid prescription 4 to 90 days after discharge and 1 or more prescription 91 to 365 days after discharge among women who filled peripartum opioid prescriptions.

**Results:**

In total, 308 226 deliveries were included: 195 013 (63.3%) vaginal deliveries and 113 213 (36.7%) cesarean deliveries. Participant mean (SD) age was 31.3 (5.3) years, and 70 567 (51.0%) were white patients. Peripartum opioid prescriptions were filled by 27.0% of women with vaginal deliveries and 75.7% of women with cesarean deliveries. Among them, 1.7% of those with vaginal deliveries and 2.2% with cesarean deliveries had new persistent opioid use. By contrast, among women not receiving a peripartum opioid prescription, 0.5% with vaginal delivery and 1.0% with cesarean delivery had new persistent opioid use. From 2008 to 2016, opioid prescription fills decreased for vaginal deliveries from 26.9% to 23.8% (*P* < .001) and for cesarean deliveries from 75.5% to 72.6% (*P* < .001), and fewer women had new persistent use (vaginal delivery, from 2.2% to 1.1%; *P* < .001; cesarean delivery, from 2.5% to 1.3%; *P* < .001). The strongest modifiable factor associated with new persistent opioid use after delivery was filling an opioid prescription before delivery (adjusted odds ratio, 1.40; 95% CI, 1.05-1.87). For vaginal deliveries, receiving a prescription equal to or more than 225 oral morphine equivalents was associated with new persistent opioid use (adjusted odds ratio, 1.25; 95% CI, 1.06-1.48). Women who underwent cesarean delivery and had a hysterectomy were more likely to develop persistence (AOR, 2.75; 95% CI, 1.33-5.70), although women who underwent a nonelective (AOR, 0.97; 95% CI, 0.88-1.07) or repeat cesarean (AOR, 1.45; 95% CI, 0.93-2.28) were not more likely. For cesarean deliveries, risk factors were associated with patient attributes such as tobacco use (adjusted odds ratio, 1.82; 95% CI, 1.56-2.11), psychiatric diagnoses, history of substance use (adjusted odds ratio, 1.43; 95% CI, 1.10-1.86), and pain conditions.

**Conclusions and Relevance:**

The results of the present study suggested that opioid prescribing and new persistent use after vaginal delivery or cesarean delivery have decreased since 2008. However, modifiable prescribing patterns were associated with persistent opioid use for patients who underwent vaginal delivery, and risk factors following cesarean delivery mirrored those of other surgical conditions. Judicious opioid prescribing and preoperative risk screening may be opportunities to decrease new persistent opioid use after childbirth.

## Key Points

**Question:**

What are the rates of new persistent opioid use among women who receive an opioid prescription after undergoing vaginal or cesarean delivery?

**Findings:**

In this US national cohort study of 308 226 deliveries, women who received a peripartum opioid prescription had rates of new persistent opioid use of 1.7% for vaginal delivery and 2.2% for cesarean delivery. Prescription size and filling a prescription before delivery were associated with new persistent opioid use.

**Meaning:**

These results suggest that maternity care clinicians can potentially decrease new persistent opioid use among women after either vaginal or cesarean delivery through judicious opioid prescribing.

## Teaser

This cohort study uses US national insurance claims data from a single private payer to assess the association between opioid prescribing before and after vaginal or cesarean delivery and the rates of new persistent opioid use among these women.

## Introduction

Maternity care is the most common reason for hospitalization in the United States, with 3.86 million births annually.^1,2^ More than one-third of women who give birth each year have a cesarean delivery, two-thirds of whom receive a peripartum opioid prescription.^1,3^ Two-thirds of women who give birth each year will have a vaginal delivery, of whom approximately one-quarter will receive an opioid prescription.^1,4^

The risk of new persistent opioid use among opioid-naive patients after a perioperative opioid prescription is well described across a variety of procedural-based specialties.^5,6,7^ Notably, patient factors and prescribing patterns account for a substantial proportion of the risk of developing new persistent use. For example, recent data indicate that there is no difference in this risk between major and minor surgical procedures.^5^ These data suggest that it is the opioid exposure itself, not the magnitude of the procedure, that is associated with persistent opioid use.^5^

Postoperative opioid prescribing has been shown to increase the risk of new persistent opioid use after cesarean delivery, but definitions used in obstetrics have been more stringent than those used in other surgical specialties and thus may underestimate rates.^3,5^ In addition, rates of new persistent opioid use after vaginal delivery have not been defined on a national level.^8^

Because more than 1 million women will fill a peripartum opioid prescription each year, understanding opioid prescribing after pregnancy is important for minimizing opioid harms.^1,3,4^ We sought to describe rates and trends of postdelivery opioid prescribing and new persistent opioid use in a national cohort of women who underwent a vaginal or cesarean delivery. In addition, we describe the factors associated with new persistent opioid use in this population.

## Methods

### Study Design

In this retrospective cohort study, we used data from Clinformatics Data Mart (Optuminsight), which aggregates claims from a single large, national private payer and includes medical and prescription drug coverage. We examined all claims from 2008 to 2016 for women who underwent a vaginal or cesarean delivery as identified by *International Classification of Diseases, Ninth Revision* (*ICD*-*9*) or *International Statistical Classification of Diseases and Related Health Problems, Tenth Revision* (*ICD-10*) procedure codes or *Current Procedural Terminology* (*CPT*) codes (vaginal delivery: *ICD-9* codes V27.x and 650, or 654.20; *ICD-10* codes O80x or O34.219; *CPT* codes V27.x and 59400, 59409, 59410, 59610, 59612, or 59614; cesarean delivery: *ICD-9* codes 74.0, 74.1, 74.2, 74.4, 74.9, or 74.99; *ICD-10* code O82x but excluding O82.2; and *CPT* codes 59510, 59514, 59515, 59618, 59620, or 59622). Our goal was to assess peripartum opioid prescribing rates among all women undergoing vaginal or cesarean delivery. Thus, all reproductive age women (ages 12-55 years) were included, and delivery was the primary unit of analysis. If patients had multiple deliveries in the specified period, only the first birth was included. To ensure adequate capture of comorbidities and opioid use prior to delivery, women were included only if they had continuous medical and prescription drug enrollment from 1 year before delivery to 1 year after discharge. Women were excluded if they were not opioid naive (ie, filled an opioid prescription in the year before delivery)^5^ to best understand the independent association of peripartum prescribing. Women were also excluded if they had an index hospitalization stay of more than 30 days because such deliveries were not thought to represent routine care. In addition, we excluded patients who underwent additional procedures in the year after discharge as identified by anesthesia procedural codes. The University of Michigan Institutional Review Board (Ann Arbor) deemed the present study exempt from formal review and also waived the need to obtain patient informed consent, both because the data set was deidentified. This study followed the Strengthening the Reporting of Observational Studies in Epidemiology (STROBE) reporting guideline for cohort studies.^9^

### Exposures

The primary exposure in this study was the receipt of a peripartum opioid prescription, defined as a prescription received 1 week before delivery to 3 days after delivery. This definition has been used in other studies to account for patients receiving a preoperative prescription for postoperative pain control or delaying their initial prescription fill until after discharge.^10^ Prescription characteristics, including whether the prescription was filled before or after delivery and prescription size measured in oral morphine equivalents (OMEs), were also included.

### Main Outcome

The primary outcome in this study was new persistent opioid use, defined as a pharmacy claim for 1 or more opioid prescriptions between 4 and 90 days after discharge and 1 or more prescriptions between 91 and 365 days after discharge among women who filled a peripartum opioid prescription (1 week prior to admission and 3 days after discharge).^5^ Prescriptions were required both 4 to 90 days and 91 to 365 days after discharge to demonstrate persistent use vs a prescription fill only after 91 days, which is more likely to represent a new prescribing episode.

### Patient Factors

Several clinical and sociodemographic covariates that have previously been associated with new persistent opioid use^5,6,10,11^ were incorporated into our model, including individual-level factors as identified in claims data (age, race/ethnicity, educational level, pain disorders, and psychiatric disorders), pregnancy factors (pregnancy comorbidity index^12^ and delivery type), and contextual factors (region and year of delivery). When modeling vaginal and cesarean delivery separately, we included delivery-specific conditions (captured by *CPT*, *ICD-9*, and *ICD-10* codes) as covariates. For vaginal delivery, we included whether the patient had a vaginal birth after cesarean, prolonged labor, a higher-order perineal laceration (defined as a third- or fourth-degree laceration), and whether an operative vaginal delivery was performed (defined as a vacuum- or forceps-assisted delivery).

For cesarean delivery, we included whether the procedure was scheduled or unscheduled, whether it was the first or a repeat cesarean delivery, and whether a hysterectomy was also performed at the time of delivery.

### Statistical Analysis

We used descriptive statistics to define rates of opioid prescribing and of new persistent opioid use. Cochran-Armitage trend tests were performed to assess rates of opioid prescribing and new persistent opioid use over time. Multivariable logistic regression was used to estimate the factors associated with new persistent opioid use after vaginal delivery, cesarean delivery, and in a combined model. Missing data were reported in separate categories in regression models, with the highest rates of missing data for race/ethnicity (24%) and income (23%). All other categories had less than 5% missing data. Analyses were performed using SAS, version 9.4 (SAS Institute Inc). Statistical significance was set at *P* < .05 with 2-sided tests.

## Results

**Figure 1.**
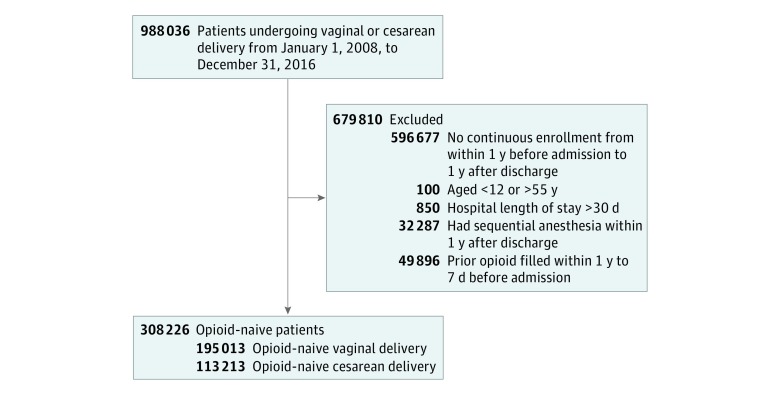
Flowchart of Patient Inclusions and Exclusions Only the first delivery was included for patients with more than 1 delivery.

We identified 988 036 women who had a vaginal or cesarean delivery in our study period. However, 596 677 women did not have continuous enrollment from 1 year before to 1 year after delivery, 100 did not meet age criteria, 850 had a length of stay longer than 30 days, 32 287 had an anesthesia event within 1 year after discharge, and 49 896 were not opioid naive (Figure 1).

Of the 308 226 eligible deliveries identified in our study period, 195 013 (63.3%) were vaginal deliveries and 113 213 (36.7%) were cesarean deliveries. The number of patients filling a peripartum opioid prescription was 52 710 (27.0%) for vaginal deliveries and 85 730 (75.7%) for cesarean deliveries. The median number of OMEs per patient was 150 (interquartile range, 112.5-225.0) for vaginal deliveries, which is equivalent to 20 tablets of oxycodone, 5 mg, and 225 (interquartile range, 150.0-241.4) for cesarean deliveries, which is equivalent to 30 tablets of oxycodone, 5 mg. In the peripartum period, 893 of 52 710 patients with vaginal delivery (1.7%) and 2633 of 85 730 patients with cesarean delivery (3.1%) filled 2 or more prescriptions.

Descriptive data are provided in Table 1. The mean (SD) age of the study cohort was 31.3 (5.3) years (vaginal delivery mean [SD] age, 30.1 [5.2] years; cesarean delivery mean [SD] age, 32.0 [5.2] years), and the majority (70 567 of 138 440 [51.0%]) were white patients (vaginal deliveries, 28 477 of 52 710 [54.0%]; cesarean deliveries, 42 090 of 85 730 [49.1%]). Patients with cesarean delivery had a longer mean (SD) length of stay (vaginal delivery, 2.3 [1.0] vs cesarean delivery, 3.5 [1.9]) and higher mean (SD) comorbidity index (vaginal delivery, 0.7 [1.1] vs cesarean delivery, 1.3 [1.5]; *P* < .001) (Table 1).

**Table 1.** Characteristics of Patients Who Underwent Vaginal or Cesarean Delivery With or Without New Persistent Opioid Use

Characteristic	Patients, No. (%)			*P* Value^a^	Patients, No. (%)			*P* Value^a^	*P* Value for Vaginal vs Cesarean Delivery^a^
	Vaginal Delivery Cohort (n = 52 710)	Persistent Opioid Use (n = 893)	No Persistent Opioid Use (n = 51 817)		Cesarean Delivery Cohort (n = 85 730)	Persistent Opioid Use (n = 1897)	No Persistent Opioid Use (n = 83 833)		
Age, y									
<20	1601 (3.0)	39 (4.4)	1562 (3.0)	<.001	1157 (1.3)	54 (2.8)	1103 (1.3)	<.001	<.001
20-29	21 521 (40.8)	422 (47.3)	21 099 (40.7)		24 734 (28.9)	645 (34.0)	24 089 (28.7)		
30-39	27 853 (52.8)	403 (45.1)	27 450 (53.0)		53 435 (62.3)	1066 (56.2)	52 369 (62.5)		
≥40	1735 (3.3)	29 (3.2)	1706 (3.3)		6404 (7.5)	132 (7.0)	6272 (7.5)		
Race/ethnicity									
White	28 477 (54.0)	486 (54.4)	27 991 (54.0)	<.001	42 090 (49.1)	997 (52.6)	41 093 (49.0)	<.001	<.001
Black	3306 (6.3)	79 (8.8)	3227 (6.2)		6676 (7.8)	199 (10.5)	6477 (7.7)		
Hispanic	4573 (8.7)	74 (8.3)	4499 (8.7)		8935 (10.4)	187 (9.9)	8748 (10.4)		
Asian	3407 (6.5)	27 (3.0)	3380 (6.5)		6643 (7.7)	60 (3.2)	6583 (7.9)		
Unknown	12 947 (24.6)	227 (25.4)	12 720 (24.5)		21 386 (24.9)	454 (23.9)	20 932 (25.0)		
Region									
Northeast	2188 (4.2)	27 (3.0)	2161 (4.2)	.008	8808 (10.3)	103 (5.4)	8705 (10.4)	<.001	<.001
Midwest	13 026 (24.7)	187 (20.9)	12 839 (24.8)		19 810 (23.1)	393 (20.7)	19 417 (23.2)		
South	26 551 (50.4)	495 (55.4)	26 056 (50.3)		40 888 (47.7)	1002 (52.8)	39 886 (47.6)		
West	10 871 (20.6)	184 (20.6)	10 687 (20.6)		16 051 (18.7)	393 (20.7)	15 658 (18.7)		
Unknown	74 (0.1)	0 (0)	74 (0.1)		173 (0.2)	6 (0.3)	167 (0.2)		
Educational level									
<12th Grade	205 (0.4)	3 (0.3)	202 (0.4)	<.001	385 (0.4)	8 (0.4)	377 (0.4)	<.001	<.001
High school diploma	10 036 (19.0)	198 (22.2)	9838 (19.0)		16 647 (19.4)	476 (25.1)	16 171 (19.3)		
<Bachelor degree	28 943 (54.9)	537 (60.1)	28 406 (54.8)		45 547 (53.1)	1040 (54.8)	44 507 (53.1)		
Bachelor degree plus	13 160 (25.0)	151 (16.9)	13 009 (25.1)		22 404 (26.1)	359 (18.9)	22 045 (26.3)		
Unknown	366 (0.7)	4 (0.4)	362 (0.7)		747 (0.9)	14 (0.7)	733 (0.9)		
Household income range, $									
<40 000	4849 (9.2)	109 (12.2)	4740 (9.1)	<.001	8225 (9.6)	261 (13.8)	7964 (9.5)	<.001	.08
40 000-49 999	2479 (4.7)	44 (4.9)	2435 (4.7)		4034 (4.7)	90 (4.7)	3944 (4.7)		
50 000-59 999	2766 (5.2)	64 (7.2)	2702 (5.2)		4575 (5.3)	124 (6.5)	4451 (5.3)		
60 000-74 999	4272 (8.1)	81 (9.1)	4191 (8.1)		6659 (7.8)	183 (9.6)	6476 (7.7)		
75 000-99 999	6298 (11.9)	100 (11.2)	6198 (12.0)		10 122 (11.8)	220 (11.6)	9902 (11.8)		
≥100 000	19 906 (37.8)	289 (32.4)	19 617 (37.9)		32 299 (37.7)	554 (29.2)	31 745 (37.9)		
Unknown	12 140 (23.0)	206 (23.1)	11 934 (23.0)		19 816 (23.1)	465 (24.5)	19 351 (23.1)		
Delivery year									
2008	7594 (14.4)	168 (18.8)	7426 (14.3)	<.001	12 418 (14.5)	308 (16.2)	12 110 (14.4)	<.001	<.001
2009	7277 (13.8)	128 (14.3)	7149 (13.8)		12 522 (14.6)	337 (17.8)	12 185 (14.5)		
2010	6751 (12.8)	151 (16.9)	6600 (12.7)		11 185 (13.0)	276 (14.5)	10 909 (13.0)		
2011	6158 (11.7)	118 (13.2)	6040 (11.7)		9764 (11.4)	257 (13.5)	9507 (11.3)		
2012	6001 (11.4)	92 (10.3)	5909 (11.4)		9479 (11.1)	209 (11.0)	9270 (11.1)		
2013	5197 (9.9)	89 (10.0)	5108 (9.9)		7908 (9.2)	150 (7.9)	7758 (9.3)		
2014	5043 (9.6)	57 (6.4)	4986 (9.6)		7562 (8.8)	140 (7.4)	7422 (8.9)		
2015	4700 (8.9)	47 (5.3)	4653 (9.0)		7584 (8.8)	127 (6.7)	7457 (8.9)		
2016	3989 (7.6)	43 (4.8)	3946 (7.6)		7308 (8.5)	93 (4.9)	7215 (8.6)		
Hospital length of stay, d									
≤3	50 792 (96.4)	856 (95.9)	49 936 (96.4)	.10	51 821 (60.4)	1198 (63.2)	50 623 (60.4)	.04	<.001
4-7	1765 (3.3)	31 (3.5)	1734 (3.3)		32 414 (37.8)	664 (35.0)	31 750 (37.9)		
>7	153 (0.3)	6 (0.7)	147 (0.3)		1495 (1.7)	35 (1.8)	1460 (1.7)		
Used tobacco	3087 (5.9)	86 (9.6)	3001 (5.8)	<.001	5008 (5.8)	209 (11.0)	4799 (5.7)	<.001	.91
Mental health disorder									
Adjustment disorder	1101 (2.1)	33 (3.7)	1068 (2.1)	<.001	1909 (2.2)	66 (3.5)	1843 (2.2)	<.001	.09
Anxiety	2678 (5.1)	83 (9.3)	2595 (5.0)	<.001	4238 (4.9)	163 (8.6)	4075 (4.9)	<.001	.26
Mood disorder	2477 (4.7)	81 (9.1)	2396 (4.6)	<.001	3970 (4.6)	181 (9.5)	3789 (4.5)	<.001	.56
Suicide and intentional self-inflicted injury	56 (0.1)	0 (0)	56 (0.1)	>0.99	62 (0.1)	5 (0.3)	57 (0.1)	.002	.04
Personality disorders	44 (0.1)	3 (0.3)	41 (0.1)	.008	58 (0.1)	2 (0.1)	56 (0.1)	.37	.29
Attention-deficit, conduct, impulse control, and disruptive behavior disorders	745 (1.4)	30 (3.4)	715 (1.4)	<.001	889 (1.0)	47 (2.5)	842 (1.0)	<.001	<.001
Schizophrenia and other psychotic disorders	36 (0.1)	1 (0.1)	35 (0.1)	.46	78 (0.1)	4 (0.2)	74 (0.1)	.10	.15
Drug and substance use disorders	838 (1.6)	38 (4.3)	800 (1.5)	<.001	1512 (1.8)	65 (3.4)	1447 (1.7)	<.001	.02
Other mental problem	952 (1.8)	25 (2.8)	927 (1.8)	.02	1699 (2.0)	66 (3.5)	1633 (1.9)	<.001	.02
Pain disorder									
Arthritis	8625 (16.4)	200 (22.4)	8425 (16.3)	<.001	15 054 (17.6)	453 (23.9)	14 601 (17.4)	<.001	<.001
Back	7634 (14.5)	178 (19.9)	7456 (14.4)	<.001	12 040 (14.0)	380 (20.0)	11 660 (13.9)	<.001	.02
Neck	2795 (5.3)	63 (7.1)	2732 (5.3)	.02	4663 (5.4)	140 (7.4)	4523 (5.4)	<.001	.27
Other	7214 (13.7)	178 (19.9)	7036 (13.6)	<.001	12 618 (14.7)	412 (21.7)	12 206 (14.6)	<.001	<.001
Total dose tertile, mg of OME during perioperative period									
<150	17 832 (33.8)	255 (28.6)	17 577 (33.9)	.002	6993 (8.2)	146 (7.7)	6847 (8.2)	.32	<.001
150 to <225	18 762 (35.6)	329 (36.8)	18 433 (35.6)		23 971 (28.0)	508 (26.8)	23 463 (28.0)		
≥225	16 116 (30.6)	309 (34.6)	15 807 (30.5)		54 766 (63.9)	1243 (65.5)	53 523 (63.8)		
Filled opioid prescription before surgery	582 (1.1)	20 (2.2)	562 (1.1)	.001	1076 (1.3)	30 (1.6)	1046 (1.3)	.20	.01
Comorbidity index									
0	33 054 (62.7)	525 (58.8)	32 529 (62.8)	.19	34 943 (40.8)	720 (38.0)	34 223 (40.8)	.21	<.001
1	10 548 (20.0)	199 (22.3)	10 349 (20.0)		22 105 (25.8)	507 (26.7)	21 598 (25.8)		
2	5782 (11.0)	107 (12.0)	5675 (11.0)		14 510 (16.9)	345 (18.2)	14 165 (16.9)		
3	1907 (3.6)	35 (3.9)	1872 (3.6)		7104 (8.3)	169 (8.9)	6935 (8.3)		
4	739 (1.4)	17 (1.9)	722 (1.4)		3466 (4.0)	75 (4.0)	3391 (4.0)		
≥5	680 (1.3)	10 (1.1)	670 (1.3)		3602 (4.2)	81 (4.3)	3521 (4.2)		
Delivery complication									
Vaginal delivery									
VBAC	816 (1.5)	16 (1.8)	800 (1.5)	.55	NA	NA	NA	NA	NA
Bilateral tubal ligation	1361 (2.6)	40 (4.5)	1321 (2.5)	<.001	NA	NA	NA	NA	NA
Higher-order perineal laceration	4515 (8.6)	60 (6.7)	4455 (8.6)	.047	NA	NA	NA	NA	NA
Operative vaginal delivery	5897 (11.2)	108 (12.1)	5789 (11.2)	.39	NA	NA	NA	NA	NA
Prolonged labor	723 (1.4)	16 (1.8)	707 (1.4)	.28	NA	NA	NA	NA	NA
Cesarean delivery									
Peripartum hysterectomy	NA	NA	NA	NA	134 (0.2)	8 (0.4)	126 (0.2)	.01	NA
Admit type nonelective	NA	NA	NA	NA	30 732 (35.8)	696 (36.7)	30 036 (35.8)	.70	NA
Repeated cesarean delivery	NA	NA	NA	NA	682 (0.8)	20 (1.1)	662 (0.8)	.20	NA

Abbreviations: NA, not applicable; OME, oral morphine equivalent; VBAC, vaginal birth after cesarean.

^a^ Using χ^2^ or Fisher exact test when 25 of the cells have expected counts less than 5.

**Figure 2.**
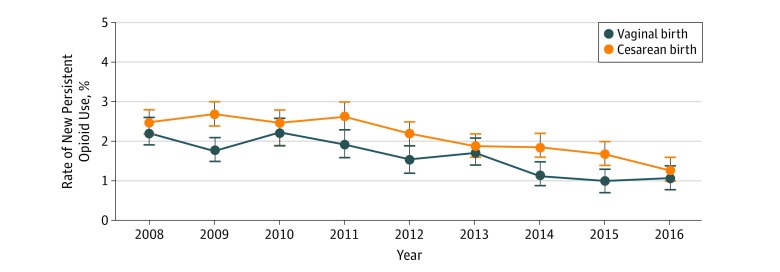
Rates of New Persistent Opioid Use for Vaginal or Cesarean Delivery Over Time Data points indicate mean values, and error bars indicate SD.

From 2008 to 2016, the incidence of opioid fills decreased for vaginal delivery (from 26.9% to 23.8%; *P* < .001) and for cesarean delivery (from 75.5% to 72.6%; *P* < .001). There were similar decreases in the number of women with new persistent opioid use during the study period (vaginal delivery, from 2.2% to 1.1%; *P* < .001; cesarean delivery, from 2.5% to 1.3%; *P* < .001) (Figure 2). Women who underwent vaginal or cesarean delivery and did not fill a peripartum opioid prescription had a new persistent opioid use rate of 0.5% for vaginal delivery and 1.0% for cesarean delivery, with an overall persistence incidence rate of 0.6% for all women.

In the combined vaginal and cesarean delivery model, women were more likely to develop new persistent opioid use if they received prescriptions before delivery admission (adjusted odds ratio [AOR], 1.40; 95% CI, 1.05-1.87) and received equal to or greater than 225 OMEs (top quartile) (AOR, 1.17; 95% CI, 1.04-1.31). Patient factors associated with new persistent opioid use included age less than 20 years (AOR, 1.32; 95% CI, 1.06-1.64), black race/ethnicity (AOR, 1.19; 95% CI, 1.04-1.36), tobacco use (AOR, 1.71; 95% CI, 1.51-1.94), and concurrent psychiatric and pain diagnoses, such as history of substance use disorder (AOR, 1.61; 95% CI, 1.30-1.98) and back pain (AOR, 1.26; 95% CI, 1.12-1.41) (Table 2). Cesarean delivery (AOR, 1.33; 95% CI, 1.21-1.46), delivering in the West region vs the Northeast region (AOR, 2.04; 95% CI, 1.68-2.48), and more recent delivery year (AOR for 2016 vs 2008, 0.46; 95% CI, 0.38-0.56) were also associated with new persistent opioid use.

**Table 2.** Multivariable Logistic Regression Models of New Persistent Opioid Use After Delivery for Vaginal, Cesarean, or Combined Delivery Models

Characteristic	Vaginal Delivery (n = 52 710)		Cesarean Delivery (n = 85 730)		Combined Vaginal and Cesarean Delivery (n = 138 440)	
	AOR (95% CI)	*P* Value	AOR (95% CI)	*P* Value	AOR (95% CI)	*P* Value
Hospital length of stay	1.06 (1.01-1.11)	.03	0.99 (0.97-1.02)	.48	1.00 (0.98-1.03)	.84
Used tobacco	1.49 (1.18-1.88)	.001	1.82 (1.56-2.11)	<.001	1.71 (1.51-1.94)	<.001
Psychiatric diagnoses, disorder						
Adjustment	1.53 (1.07-2.20)	.02	1.30 (1.00-1.67)	.049	1.37 (1.11-1.69)	.003
Anxiety	1.45 (1.12-1.88)	.004	1.37 (1.15-1.64)	.001	1.39 (1.20-1.62)	<.001
Mood	1.41 (1.09-1.84)	.01	1.63 (1.37-1.95)	<.001	1.56 (1.35-1.80)	<.001
Substance use	2.04 (1.43-2.92)	<.001	1.43 (1.10-1.86)	.008	1.61 (1.30-1.98)	<.001
Pain condition						
Arthritis	1.26 (1.04-1.52)	.02	1.23 (1.08-1.39)	.002	1.23 (1.11-1.37)	<.001
Back	1.24 (1.02-1.51)	.04	1.27 (1.10-1.45)	.001	1.26 (1.12-1.41)	<.001
Neck	0.99 (0.74-1.32)	.96	0.99 (0.81-1.20)	.90	0.99 (0.84-1.16)	.88
Other	1.29 (1.08-1.54)	.005	1.37 (1.22-1.54)	<.001	1.35 (1.22-1.49)	<.001
Total dose tertile, mg of OME during perioperative period						
<150	1 [Reference]	NA	1 [Reference]	NA	1 [Reference]	NA
150 to <225	1.22 (1.03-1.44)	.02	0.99 (0.82-1.19)	.90	1.12 (0.99-1.27)	.07
≥225	1.25 (1.06-1.48)	.009	1.04 (0.87-1.24)	.66	1.17 (1.04-1.31)	.01
Comorbidity index	1.02 (0.96-1.09)	.48	1.03 (1.00-1.07)	.05	1.03 (1.00-1.06)	.03
Filled opioid prescription before surgery	1.92 (1.22-3.03)	.005	1.19 (0.82-1.72)	.36	1.40 (1.05-1.87)	.02
Delivery complication (vaginal delivery only)						
VBAC	1.22 (0.74-2.02)	.44	NA	NA	NA	NA
Bilateral tubal ligation	1.68 (1.21-2.34)	.002	NA	NA	NA	NA
Higher-order perineal laceration	0.79 (0.60-1.04)	.09	NA	NA	NA	NA
Operative vaginal delivery	1.09 (0.88-1.35)	.45	NA	NA	NA	NA
Prolonged labor	1.45 (0.86-2.44)	.16	NA	NA	NA	NA
Delivery complication (cesarean delivery only)						
Peripartum hysterectomy	NA	NA	2.75 (1.33-5.70)	.006	NA	NA
Admit type						
Elective	NA	NA	1 [Reference]	NA	NA	NA
Nonelective	NA	NA	0.97 (0.88-1.07)	.59	NA	NA
Unknown	NA	NA	0.95 (0.80-1.13)	.54	NA	NA
Repeat cesarean delivery	NA	NA	1.45 (0.93-2.28)	.10	NA	NA

Abbreviations: AOR, adjusted odds ratio; NA, not applicable; OME, oral morphine equivalent; VBAC, vaginal birth after cesarean.

In the models separated by delivery type, women who underwent vaginal delivery and had a bilateral tubal ligation were more likely to develop opioid persistence (AOR, 1.68; 95% CI, 1.21-2.34), although women who had a vaginal birth after cesarean (AOR, 1.22; 95% CI, 0.74-2.02), higher-order perineal laceration (AOR, 0.79; 95% CI, 0.60-1.04), operative delivery (AOR, 1.09; 95% CI, 0.88-1.35), or prolonged labor (AOR, 1.45; 95% CI, 0.86-2.44) were not more likely to develop opioid persistence. Receiving a prescription prior to delivery (AOR, 1.92; 95% CI, 1.22-3.03) and prescription equal to or greater than 225 OMEs (top quartile) (AOR, 1.25; 95% CI, 1.06-1.48) were factors associated with new persistent opioid use. Patient factors, including tobacco use (AOR, 1.49; 95% CI, 1.18-1.88), psychiatric diagnoses, history of substance use (AOR, 2.04; 95% CI, 1.43-2.92), and pain conditions were also associated with new persistent opioid use (Table 2).

Women who underwent cesarean delivery and had a hysterectomy were more likely to develop persistence (AOR, 2.75; 95% CI, 1.33-5.70), although women who underwent a nonelective (AOR, 0.97; 95% CI, 0.88-1.07) or repeat cesarean (AOR, 1.45; 95% CI, 0.93-2.28) were not more likely. Neither receiving a daily dose equal to or greater than 225 OMEs (top quartile) (AOR, 1.04; 95% CI, 0.87-1.24) nor receiving a prescription before delivery (AOR, 1.19; 95% CI, 0.82-1.72) were associated with new persistent opioid use. Patient factors, including tobacco use (AOR, 1.82; 95% CI, 1.56-2.11), psychiatric diagnoses, history of substance use (AOR, 1.43; 95% CI, 1.10-1.86), and pain conditions were also associated with new persistent opioid use (Table 2).

## Discussion

Among our cohort of privately insured women giving birth in the United States, more than 25% of women with vaginal deliveries and 75% of women with cesarean deliveries received a peripartum opioid prescription. Of those women, 1% to 2% continued to fill opioids for months following birth. Encouragingly, rates of prescribing and new persistent opioid use decreased over time, although these were relatively modest decreases. These changes are consistent with recent data that demonstrate rates of opioid prescribing in the United States have decreased over time.^13,14,15^ Individual, pregnancy, and contextual factors were associated with increased risk of new persistent opioid use; although prescription factors were not associated with persistence after cesarean delivery, prescription timing and prescription size were the largest and most modifiable factors associated with developing new persistent opioid use after vaginal delivery.

### New Persistent Opioid Use After Delivery

Based on the present findings, roughly 77 000 of the 3.86 million women who deliver each year are at risk of continued opioid use.^1,3,4^ Other studies using narrower definitions that may underestimate opioid use have shown more modest rates of persistent opioid use after cesarean delivery.^3,8^ A prior nationwide study of private claims data identified a rate of opioid persistence for 1 of 300 in opioid-naive women undergoing cesarean delivery based on trajectory models, with patients in the persistent group filling at least 1 prescription per month from 6 months to 1 year after delivery.^3^ Similarly, a recent study of women enrolled in Medicaid giving birth in Tennessee found a new persistent opioid use rate of 0.84% after cesarean delivery and of 0.59% after vaginal delivery, defining prolonged use as a prescription fill once every 45 days from 6 weeks postpartum to 1 year after birth.^8^

Despite the differences in the nuances of defining new persistent opioid use, our findings highlight that a substantial number of patients continue to fill opioid prescriptions long after expected recovery. These definitions have been used in the surgical literature to highlight inappropriate continued opioid prescribing.^11^ This prolonged use may be particularly concerning in a postpartum cohort, in which women are caring not only for themselves but also for a newborn.^16^ Future work will need to clarify under what conditions patients are receiving opioids as well as where and from whom they are receiving prescriptions to develop effective strategies to mitigate the risk of conversion to long-term opioid use.

### Role of Opioid Prescribing and Persistence

The association between perioperative opioid prescribing and new persistent opioid use has been well documented in the literature.^5,6,10,11^ Previous studies have suggested that postoperative pain is not the only factor contributing to persistence because new persistent opioid use is similar between major and minor surgical procedures despite differing levels of anticipated pain.^5^ The rates of new persistent opioid use after wisdom tooth extraction, another procedure commonly performed in a young, otherwise healthy population, are similar to those found in the present study: 1.3% vs 2.0%.^10^ The difference in rates of new persistent use in the present study after vaginal (1.7%) and cesarean delivery (2.2%), although statistically significant, was small, consistent with prior findings that opioid prescribing, rather than the magnitude of the procedure, was associated with new persistent opioid use.

The prescription characteristics most associated with new persistent opioid use in prior studies of other surgical procedures have included prescription timing and prescription size.^5,6,10^ In our study, receiving a prescription prior to admission and the number of OMEs were both associated with new persistent opioid use for vaginal delivery. By contrast, prescription characteristics were not associated with new persistent opioid use after cesarean delivery. These findings are consistent with prior studies, which have shown no association between prescription size or number of days and persistent use after cesarean delivery, and match the risk factors identified for other surgical specialties, such as tobacco use, psychiatric diagnoses (including history of substance abuse), and pain conditions.^3,5,6^ Still, the rate of new persistent opioid use following cesarean delivery was more than twice as high among women who received a peripartum prescription than among those who did not, emphasizing the importance of careful and limited prescribing during this time.

Nearly 50 000 women in our initial cohort were excluded for not being opioid naive in the year before pregnancy. Prior studies have found that between 7% and 21% of patients will receive an opioid prescription during pregnancy, but little is known about the association of this prescribing, that is, whether receipt of an opioid during pregnancy is an additional risk factor for new persistent opioid use.^17,18^ Because receipt of an opioid prescription after nonsurgical encounters, such as vaginal delivery, is associated with new persistent use, understanding the association of filling other opioid prescriptions during pregnancy will be crucial for counseling women and informing best practices. Similarly, rates of new persistent opioid use after nonprocedural acute pain conditions, such as kidney stones and nonoperative fractures, have not been well described and could be important for defining guidelines for prescribing.

We identified significant regional differences in new persistent opioid use that match previously identified variations in prescribing following delivery.^19,20,21^ Opioid prescribing after uncomplicated delivery has been shown to vary 7-fold by census tract for patients with Medicaid.^22^ Variation in health care delivery is a known quality indicator of resource use: the best available evidence shows the current rates in the United States are a marker of overprescribing for many women.^23,24^ Up to 75% of patients who undergo cesarean delivery report having unused opioid tablets at home ranging from 75 to 150 OMEs (the equivalent of 10 to 20 oxycodone tablets of 5 mg each).^19,21^ Among women with unused tablets at home, 63% to 95% have not disposed of the excess medication.^19^ These unnecessary tablets represent a risk not only to patients but also to the community through diversion if not properly managed given that 20% of patients report sharing prescription medications.^25^ Increased standardization should be considered to reduce individual and societal consequences of postpartum opioid overprescribing.

### Potential Influence of Opioid-Sparing Pain Control and Counseling

Ensuring that patients’ postpartum pain is well controlled must remain a priority.^16,26^ Opioid-sparing pain protocols for cesarean delivery have been shown to successfully decrease opioid prescribing while maintaining patient comfort.^27,28^ These protocols include 3 main components: patient preparation (when cesarean delivery is scheduled), perioperative measures, and multimodal postoperative therapy. Preparation can help patients set expectations and develop coping techniques prior to surgery.^29^ Intraoperative measures, including preoperative acetaminophen and intrathecal long-acting opioids with regional anesthetic, have been shown to decrease postoperative pain.^30,31^ Similarly, using scheduled nonnarcotic medications, such as acetaminophen and ketorolac or ibuprofen, has been shown to reduce the total OME requirement per patient.^27,28^ Enhanced Recovery After Surgery protocols for obstetrics have the potential to coordinate pain management across the delivery episode through standardized protocols.^30,31,32^ However, there is a lack of data on appropriate pain management techniques following vaginal delivery, and more work is needed to understand the role of opioids and opioid-sparing pain protocols for both vaginal delivery and cesarean delivery.^33^

Reducing postpartum discharge prescribing may also be possible through tailored prescribing and shared decision-making.^27,34,35^ Tailoring patients’ discharge prescriptions based on their inpatient opioid use has been shown to decrease the number of opioid tablets prescribed by more than 50% (30 vs 14 tablets) without changing patient self-reported pain outcomes.^34^ Shared decision-making about the number of opioid tablets prescribed at discharge combined with patient counseling about pain control has led to similar decreases in prescription size, with an overall decrease of 35% (33 vs 22 tablets).^27^ More recently, opioid-free protocols have been used following cesarean delivery, with more than 50% of enrolled patients able to navigate the postpartum period without any opioids.^36^

### Need for Better Guidelines

Current guidelines on postpartum prescribing are vague and do not provide specific recommendations on appropriate prescribing following vaginal delivery or cesarean delivery.^16^ The guidelines from both the American College of Obstetrics and Gynecology and the American Pain Society advocate for stepwise, multimodal approaches to pain management, including shared decision-making with patients about opioid use.^16,37^ Still, neither guideline includes specific recommendations on whether women should be discharged with an opioid prescription after vaginal delivery or cesarean delivery and, if so, with how many tablets. This lack of guidance is attributable in part to a lack of sufficient data to guide recommendations: whereas the median discharge prescription OMEs in our cohort was 150 and 225 for vaginal delivery and cesarean delivery, respectively, other protocols have shown significant decreases in prescription size without increasing refill rates or worsening patients’ pain.^27,34,35^ Furthermore, according to a survey of European anesthesiologists, European women “almost never” receive an opioid prescription at discharge after vaginal birth or cesarean birth, suggesting current practices in the United States represent overprescribing.^38^ Future work will need to clarify appropriate limits and how broader implementation of opioid-sparing (or opioid-free) protocols, tailored prescriptions, and shared decision-making at discharge can influence patients’ opioid requirements and pain control after vaginal delivery or cesarean delivery.^33^

While society guidelines remain vague, 22 states have enacted opioid-prescribing regulations limiting high morphine equivalent daily dosing via a variety of policy types, including guidelines, regulations, and prior authorizations.^39,40^ The association of these different approaches with new persistent opioid use has yet to be fully seen, and it remains uncertain which policy levers will best minimize both opioid and patient harms. Still, with increasing regulation, maternity care clinicians may be forced to consider alternative approaches to pain control even before society guidelines dictate change.^41^

### Limitations

Although we believe that this study fills important knowledge gaps about peripartum opioid use, it does have several limitations. First, we used a single private insurer data source, which may limit our study’s generalizability to patients with public insurance or to those who are uninsured. Still, more than half of US births are covered by commercial insurance, suggesting our results are widely applicable.^1^ Second, we were unable to capture more granular aspects of patient pregnancy and experience, such as infant complication, acute pain score, or stressful delivery. We mitigated these omissions somewhat by including the pregnancy comorbidity index^12^ and delivery complications, such as prolonged labor; however, prospective data will be needed to obtain sufficient detail. Third, we are able to identify opioid prescription fills only through outpatient pharmacy claims. We could not capture information for patients who were prescribed opioids but did not fill their prescriptions or track how many tablets were actually consumed among patients who filled prescriptions. In fact, our rates of persistent use may represent an underestimate given that the Centers for Disease Control and Prevention reports 55% to 70% of patients receive nonprescription opioids from friends and family.^25^ Fourth, we could not account for state and national policy changes, including prescribing limits, prior authorizations, or Physician Drug Monitoring Programs requirements that may have influenced prescribing behavior. Fifth, we used a more inclusive definition of new persistent opioid use than has been previously published in the obstetrics literature.^19^ Still, our definition has been used in other surgical specialties and identified an important population who continued to use opioids after expected surgical recovery.^5,6,10^

## Conclusions

Among women giving birth in the United States, new persistent opioid use was found in the present study to decrease. Nonetheless, 1% to 2% of women in our cohort continued to fill opioid prescriptions for months following birth, particularly those women who received a larger prescription or a prescription prior to delivery. Maternity care clinicians can help decrease opioid harms by identifying risk, using opioid-sparing protocols, and providing close opioid stewardship in the peripartum period.
