# Error in Discussion

**DOI:** 10.1001/jamanetworkopen.2019.11235

**Published:** 2019-08-21

In the second sentence of the fourth paragraph of the “Role of Opioid Prescribing and Persistence” subsection of the Discussion in the Original Investigation titled “Rates of New Persistent Opioid Use After Vaginal or Cesarean Birth Among US Women”^1^ published July 26, 2019, privately insured patients were misidentified as patients with Medicaid. The sentence should have read as follows: “Opioid prescribing after uncomplicated delivery has been shown to vary 7-fold by census tract for privately insured patients.” This article has been corrected.^1^
